# A new species of *Hemichela* Stock, 1954 from the South China Sea (Arthropoda, Pycnogonida, Ammotheidae)

**DOI:** 10.3897/zookeys.526.5963

**Published:** 2015-10-08

**Authors:** Jianjia Wang, Zhen Xia, Rongcheng Lin, Qianyong Liang, Heshan Lin, Jianjun Wang, Chengxing Zheng

**Affiliations:** 1 Third Institute of Oceanography, SOA, Daxue Road No.178, Xiamen, China; 2 Guangzhou Marine Geological Survey, CGS, Huanshidong Road No. 477, Guangzhou, China

**Keywords:** Deep sea, South China Sea, Pycnogonida, *Hemichela*

## Abstract

A new species of pycnogonid collected by the Chinese research vessel, R/V HY IV, during deep sea cruises to the South China Sea in 2013, is described. The new species, *Hemichela nanhaiensis*, obtained from more than 1300 m depth, is distinguished from the other two species in the genus by the characters of the chela dactylus with 12 denticulations on the inner margin and by the presence of taller tubercles on the lateral processes.

## Introduction

Stock (1954) established the new genus *Hemichela* based on the presence of a single dactylus on the chela, segregating it from the closely-related genus *Paranymphon* and he then described *Hemichela micrasterias* the type species of the new genus. According to Bamber et al. (2015) the genus includes two named species to date, *Hemichela micrasterias* Stock, 1954 and *Hemichela longiunguis* Staples, 1982, both from western Pacific localities only. *Hemichela micrasterias* has been found in Indonesia (Stock 1954; Nakamura and Child 1990), Philippines (Child 1988b) and Japan (Nakamura and Child 1991) at a depth range of 20–657 meters. *Hemichela longiunguis* is only known from Queensland, Australia, at depths shallower than 5.8 m (Staples 1982).

A re-examination of the types of both of these species by Stock (1985) corrected certain characteristics inadvertently overlooked in his initial description (Stock 1954). Nakamura and Child (1990) demonstrated differences between *Hemichela micrasterias* from the Flores Sea and the holotype, and described the sub-adult and juvenile stages of this species. Bamber (1992) summarized the localities where these species have been found, and discovered evidence for Child’s (1983, 1988a) zoogeographic ‘corridor’ from the Antipodes to Japan.

The Pycnogonida from the islands off the western Pacific coast of China have been summarized by Bamber (1992). Japanese collections were covered by Nakamura and Child (1988a, b, 1990), and Child produced a notable series on the pycnogonids from the western Pacific Islands (1982, 1983, 1988b, 1989, 1990, 1991, 1995, 1996a, b, 1998, 1999). However, despite the long coastlines and numerous islands, pycnogonids from China have been generally poorly studied. Lou (1936a, b) described the sea spiders taken from Yantai and Jiaozhou bays. Bamber (1992, 2004, 2008) and Bamber and Morton (1997) published on the pycnogonids of the South China Sea, Taiwan, and Hong Kong. Huang and Lin (2012) illustrated 13 species recorded in the seas adjacent to China. Compared with the work completed in neighboring seas as have, for example, Nakamura and Child (1991) in Japan, Hong and Kim (1987) in Korea, and Stock (1991) in the Philippines, research on the pycnogonids of China seas are still insufficient.

During 2013, research, including benthic surveying, water sampling, and grabbing for biological and geological specimens, was carried out by the Chinese research vessel R/V HY IV in a cruise to the South China Sea. At station DS06-13, one specimen of Pycnogonida was found representing a new species of *Hemichela* together with other benthic invertebrates such as *Chaetozone setosa* Malmgren, 1867 (Annelida: Polychaeta: Cirratulidae), *Pseudosphyrapus anomalus* (Sars, 1869) (Arthropoda: Malacostraca: Sphyrapodidae), *Asellus* sp. (Arthropoda: Malacostraca: Asellidae), *Eriopisella sechellensis* (Chevreux, 1901) (Arthropoda: Malacostraca: Eriopisidae) and *Grandidierella* sp. (Arthropoda: Malacostraca: Aoridae).

## Material and methods

The specimen was collected by a box-core and sorted from the other benthic fauna and sediments from Station DS06-13 and it is conserved as the holotype at the Third Institute of Oceanography, State Oceanic Administration, China (No. DS06-13-01). The specimen was drawn using a *camera lucida* and photographs were made with an Auto-montage system on a Leica M205 FA stereomicroscope. Measurements were made axially, dorsally for the trunk, laterally for the palp, proboscis and leg, and are given in millimeters.

## Systematics

### Class Pycnogonida Latreille, 1810 Order Pantopoda Gerstäcker, 1863 Suborder Eupantopodida Fry, 1978 Superfamily Ascorhynchoidea Pocock, 1904 Family Ammotheidae Dohrn, 1881 Genus *Hemichela* Stock, 1954

#### Hemichela nanhaiensis sp. n.

Taxon classificationAnimaliaPantopodaAmmotheidae

http://zoobank.org/8E0715B3-D42F-4CC1-8F4C-0FA3188A0ED8

Fig. 1

##### Material examined.

One male, holotype (DS06-13-01), Station DS06-13, South China Sea, 21.95°N 118.81 °E, 1317.5 m depth, BC, 5 May 2013.

##### Diagnosis.

**Figure 1.**
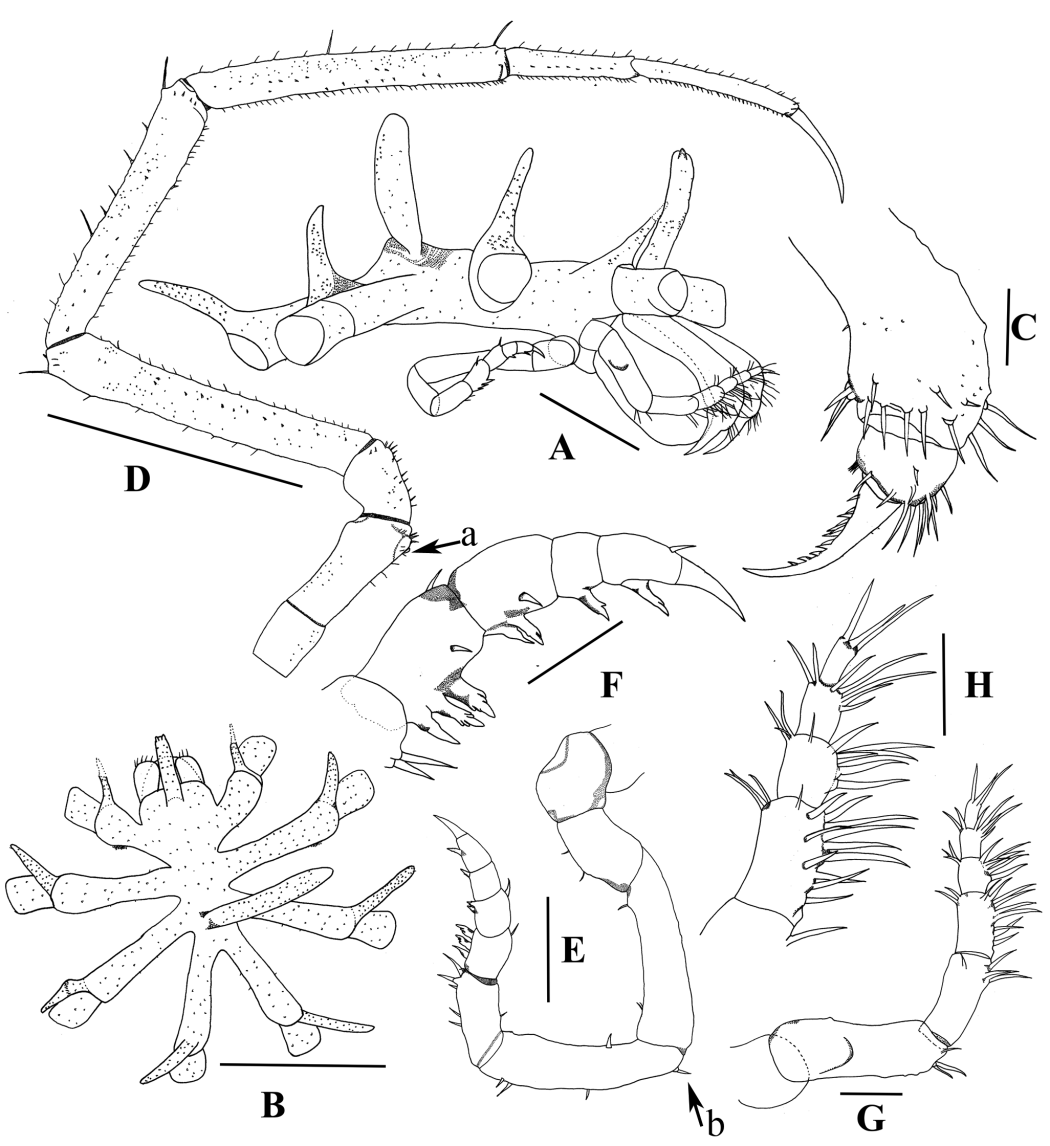
*Hemichela nanhaiensis* sp. n., DS06-13-01, male holotype: **A** trunk, lateral view **B** trunk, dorsal view **C** chela, enlarged **D** leg 3 **E** oviger **F** terminal articles of oviger, enlarged **G** palp **H** terminal articles of palp, enlarged; arrow a, position of genital pore; arrow b, reversed spine. Scale bars **A, B, D** = 1.0 mm; **C, F, H, G** = 0.1 mm; **E** = 0.2 mm.

Trunk slender, lateral processes with a single distal dorsal tubercle and armed with pedunculate asterisk-shaped setae. Ocular tubercle long with bifurcate tip (Fig. 1A, B). Chela dactylus bearing 12 denticulations on the inner margin (Fig. 1C). Palps seven-articled, second article with a conical outgrowth (Fig. 1A, G). Ovigers ten-articled, fifth article with a reversed spine located on the ventral surface near the proximal end (Fig. 1E arrow b), seventh to tenth articles with compound spines in formula 3: 2: 1: 1 (Fig. 1F). Legs slender, major articles with short lateral spines, main claw approximately 3/5 length of propodus, auxiliary claws absent (Fig. 1D).

##### Description of the holotype

**(male).** Size large for genus. Trunk slender, intersegmental lines absent, with many tiny papillae (Fig. 1A, B). Lateral processes very long, widely separated, slightly dilated distally, armed with asterisk-shaped setae on tip of tiny outgrowth. A distal dorsal tubercle is present on each lateral processe. Each tubercle long and slender, length is nearly twice diameter of lateral processes, with several minute setae. Ocular tubercle long, erected obliquely, armed with many tiny papillae, tip bifurcate with two lateral tubercles, without obvious eyes (Fig. 1A, B). Proboscis short, like a circular cone with flat tip. Abdomen very tall, directed upwards (Fig. 1A, B).

Chelifore scape one-segmented, armed distally with long setae. Palm short, with several long setae. Chela with one dactylus only, curved and bearing 12 denticulations on inner margin (Fig. 1C).

Palps seven-articled (Fig. 1G, H). First article short, without spines or setae. Second and third articles with few distal long setae. Second article longest, with a conical outgrowth on the outer surface, located in the first third of the article. Fourth to seventh articles armed with fields of ventral and distal setae mostly little longer than their article diameter (Fig. 1H).

Ovigers ten-articled (Fig. 1E, F). First and second articles stout, without setae and spines. Fourth and fifth articles longest, bearing few short setae and spines. Fifth article with a reversed spine basally. Sixth article with two distal spines and few ventral setae. Seventh to tenth articles with compound spines in formula 3: 2: 1: 1. Each spine bears one to three lateral denticulations. Terminal claw as long as tenth article (Fig. 1F).

Legs slender (Fig. 1D). Major articles with short lateral spines. First coxa short. Second coxa longest, distally swollen, with short ventral and distal seta. Genital pores present on ventral surface of second coxae of the third and fourth legs, borne on a spherical tubercle (Fig. 1D, arrow a). Third coxa short with short setae. Femur and tibiae with short ventral setae, lateral spines and long dorsal setae. Femur and second tibia subequal, longer than first tibia. Cement gland not evident. Tarsus long with short setae and spines. Propodus without heel, with single row of sole spines. Main claw strong, approximately 3/5 length of propodus. Auxiliary claws absent.

Female and juvenile are unknown.

*Measurements of holotype in mm*: Trunk length (from chelifore insertion to tip of fourth lateral processes) 3.46; width across second lateral processes 3.49; proboscis length 0.63.

Lengths of palp articles 1 to 7 respectively: 0.09; 0.25; 0.14; 0.10; 0.06; 0.05; 0.04.

Lengths of oviger articles 1 to 10 respectively: 0.06; 0.13; 0.16; 0.33; 0.33; 0.15; 0.08; 0.08; 0.06; 0.07; 0.07 (claw).

Third leg, coxa 1, 0.24; coxa 2, 0.51; coxa 3, 0.30; femur, 1.24; tibia 1, 1.13; tibia 2, 1.23; tarsus, 0.53; propodus, 0.65; claw, 0.40.

##### Etymology.

The species name, *nanhaiensis*, is derived from the Chinese language, Nanhai meaning South China Sea, referring to the location where the new species was found.

##### Remarks.

The specimen is identified as belonging to the genus *Hemichela* by the absence of a movable chela finger. Compared with *Hemichela longiunguis* and *Hemichela micrasterias*, the body size of *Hemichela nanhaiensis* is distinctly larger, length of lateral process tubercles are nearly twice diameter of lateral processes in *Hemichela nanhaiensis*, and are much taller than those in the other two species, and the *Hemichela nanhaiensis* palps are armed with more setae. The outgrowths with asterisk-shaped setae of *Hemichela micrasterias* are branching and large while the ones of *Hemichela longiunguis* and *Hemichela nanhaiensis* are simple and not distinct. The length ratio of the terminal claw and propodus, 0.62, lies between that of the previous two species (1.06 in *Hemichela longiunguis* and 0.46 in *Hemichela micrasterias*), and the relative length of the oviger terminal claw is evidently shorter than the other species in the genus (the length ratio of terminal claw and tenth article: 1 in *Hemichela nanhaiensis*, 2.33 in *Hemichela longiunguis* and 1.88 in *Hemichela micrasterias*). The chelifores are different from those of the other two species, with 12 denticulations on the inner margin of the dactylus compared to six in *Hemichela micrasterias* and two in *Hemichela longiunguis*; the chelifore scape and palm are armed with more seta than those of the other two species of the genus (Stock 1954, 1985; Staples 1982).

**Figure 2.**
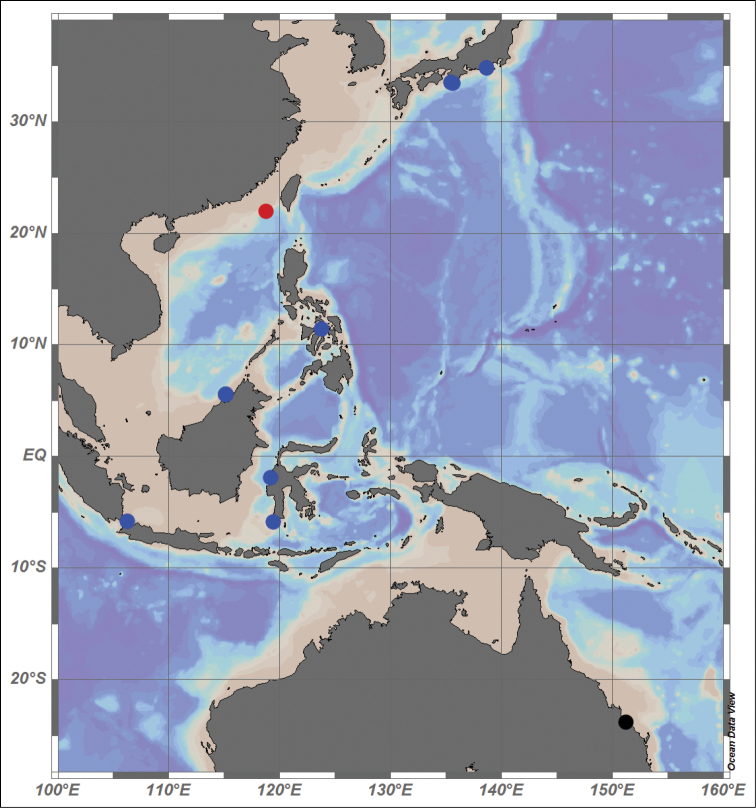
Distribution map of the three species of the genus *Hemichela*: ● *Hemichela micrasterias* ● *Hemichela nanhaiensis* ● *Hemichela longiunguis* (modified from Bamber 1992).

The records of this genus are from Japan to Queensland, Australia (Fig. 2) and the island systems of the western Pacific Ocean, with the deepest record of 657 m in the Flores Sea (Nakamura and Child 1990; Bamber 1992; Müller 1993). The new species was obtained from 1317.5 m, increasing the depth range of this genus. The occurrence of the new species fills a gap in the distribution ‘corridor’ of this genus, connecting the species in Japan with the Philippines and equatorial and Australian species.

The type habitat was sea floor predominantly composed of soft mud.

## Supplementary Material

XML Treatment for Hemichela nanhaiensis
